# Supplementary material for: Population Genetics of the Blueberry Gall Midge, Dasineura oxycoccana (Diptera: Cecidomyiidae), on Blueberry and Cranberry and Testing Invasion Scenarios
Source: Insects. 2022 Sep 28;13(10):880. doi: 10.3390/insects13100880 (PMC9604482; doi:10.3390/insects13100880)
Supplement: Supplementary file 1 [file insects-13-00880-s001.zip › Supplementary material 1 Figures S1-S4.pdf]

Population Genetics of the Blueberry Gall Midge, *Dasineura oxycoccana* (Diptera: Cecidomyiidae), on Blueberry and Cranberry and Testing Invasion Scenarios

Hyojoong Kim [1,*], Cesar Rodriguez-Saona [2,*] and Heung-Sik Lee [3]

[1] Animal Systematics Laboratory, Department of Biological Science, Kunsan National University, Gunsan, Jeonbuk 54150, Korea
[2] Department of Entomology, P.E. Marucci Center, Rutgers University, Chatsworth, NJ 08019, USA
[3] Animal & Plant Quarantine Agency, Gimcheon, Gyeongbuk 39660, Korea
* Correspondence: hkim@kunsan.ac.kr (H.K.); crodriguez@aesop.rutgers.edu (C.R.-S.)

Supplementary material 1: Figures S1-S4

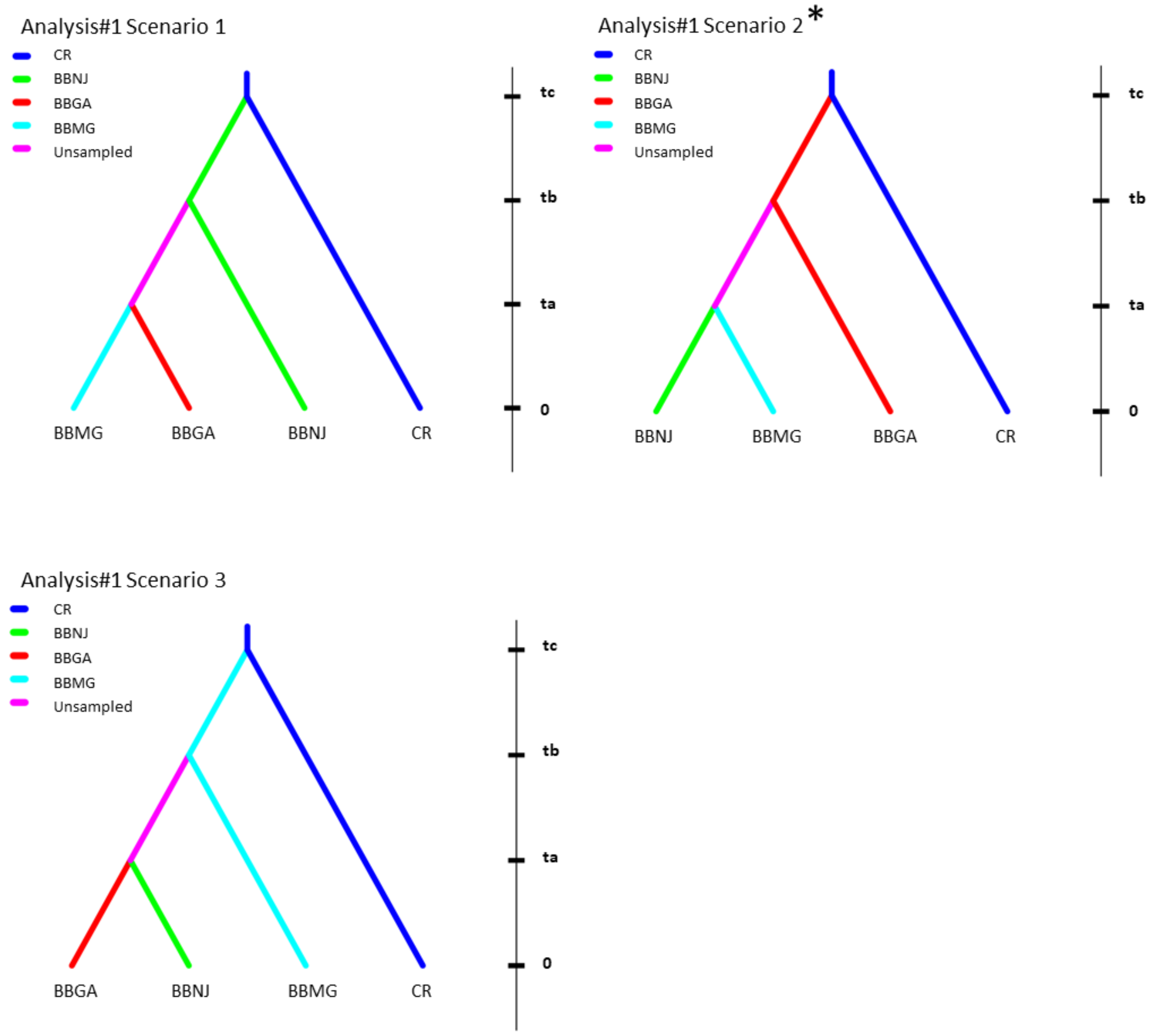

Figure S1. The simulated DIYABC historical scenarios by analysis#1. Three analysis scenarios to identify ancestral groups and to determine the divergence order of each group. Each branch's color represents a specific group, red: BBGA, BGM group from blueberry in Georgia, green: BBNJ, BGM group from blueberry in New Jersey, blue: CR, pooled CTW group from cranberry. Information of each scenario is explained in the main text.

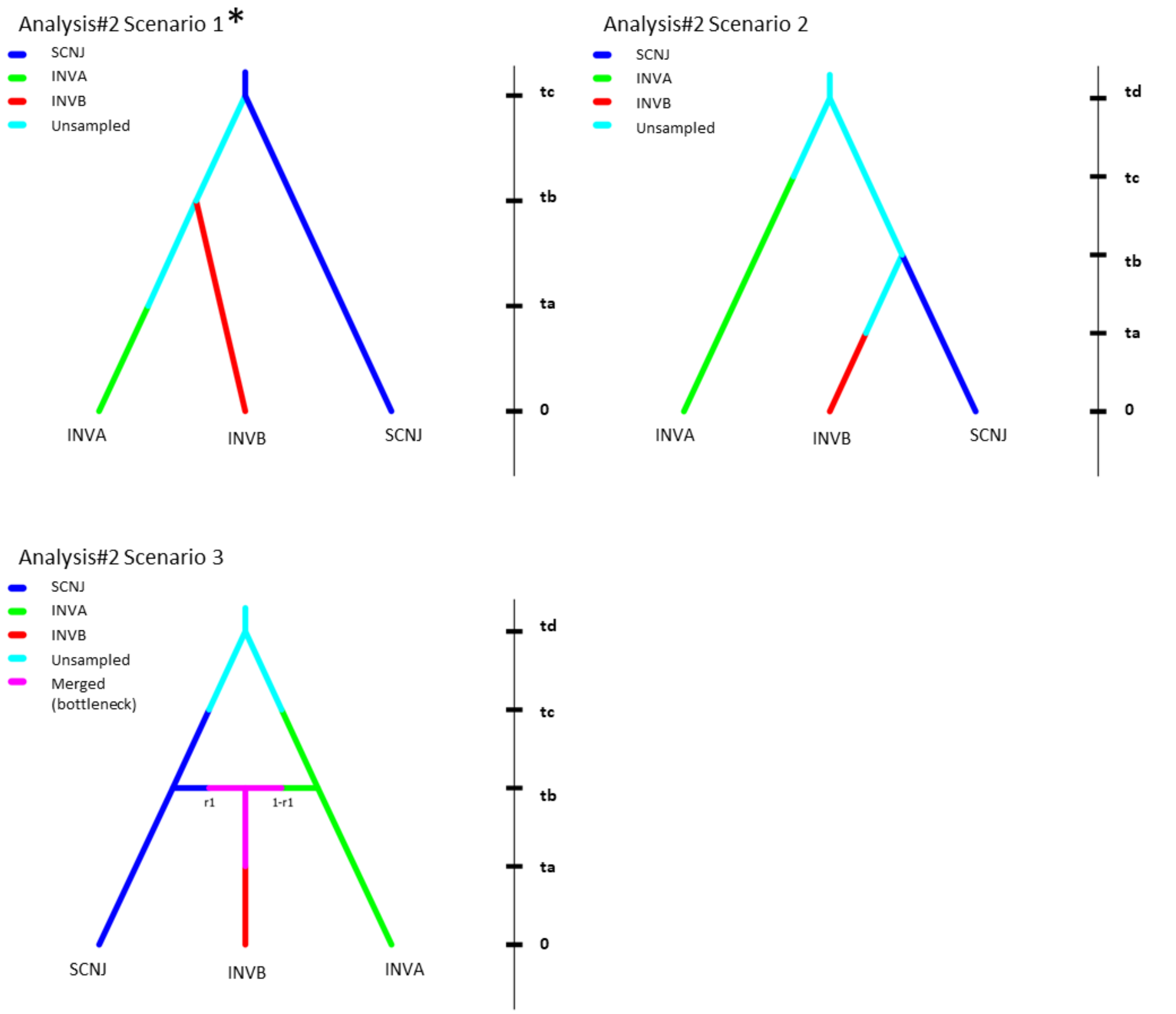

Figure S2. The simulated DIYABC historical scenarios by analysis#2. Three analysis scenarios to identify ancestral groups and to determine the divergence order of each group. Each branch's color represents a specific group, blue: SCNJ, one source blueberry-associated group, green: INVA, invasive blueberry-associated group A, red: INVB, invasive blueberry-associated group B. Information of each scenario is explained in the main text.

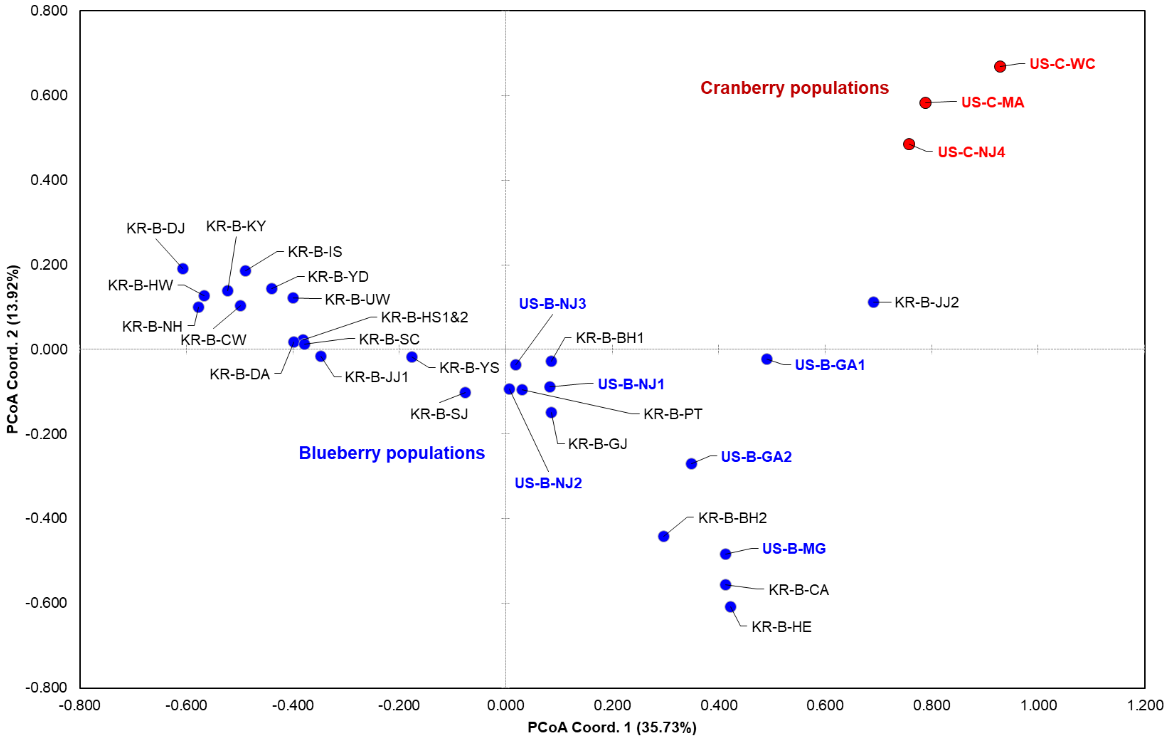

Figure S3. Principal Coordinates Analysis (PCoA) plotted by microsatellite data from 31 populations of *Dasineura oxycoccana* from cranberry and blueberry by GENALEX. The X-axis is coordinate 1 ranging from -0.80 to 1.20 and the Y-axis coordinate 2 from -0.80 to 0.80. Red circle means cranberry population, while blue blueberry one.

(a)

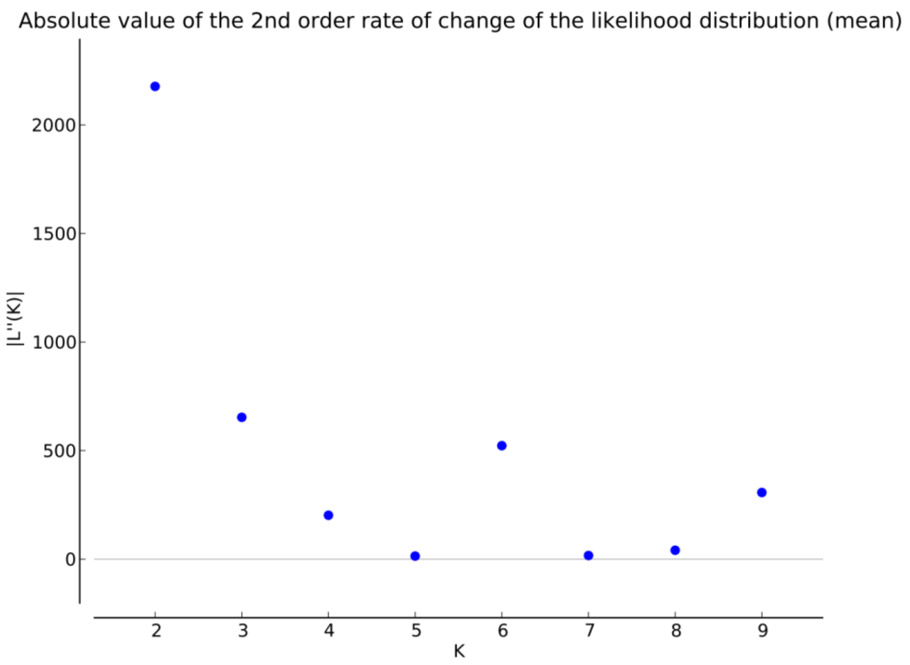

(b)

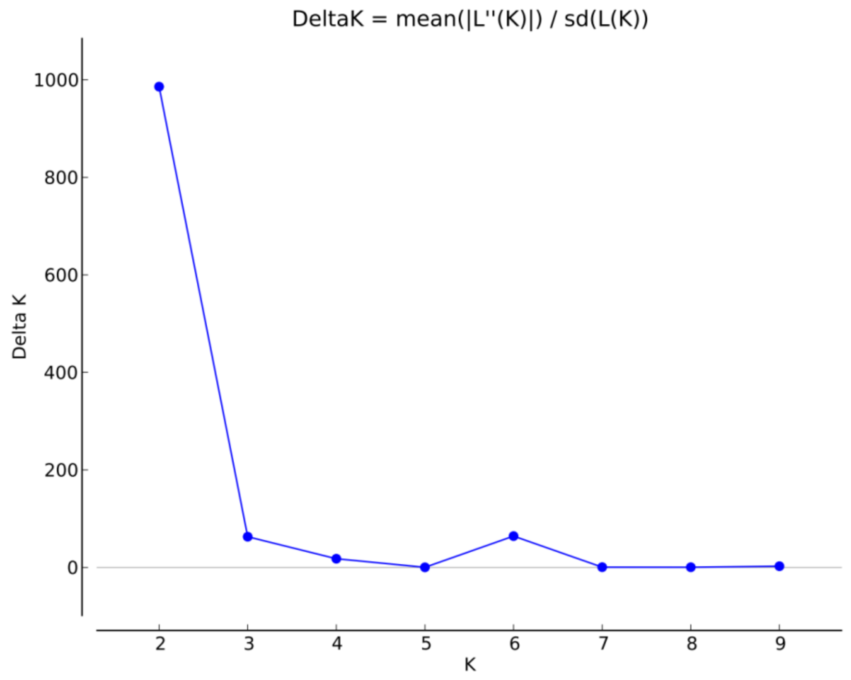

Figure S4. Results of STRUCTURE Harvester (approximate numbers). (a) Graph for the absolute value of the second order rate of change of the likelihood distribution (mean) calculated with the number of clusters (*K*) for observation of population group of *Bactrocera dorsalis*, demonstrated by the STRUCTURE Harvester (Earl 2012). Delta *K* values calculated by Evanno et al. (2005) method detecting *K* = 2 groups. (b) Mean of probabilities lnP(*K*) and their standard deviation of the posterior probability.
