# Supplementary material for: Population Genetics of the Blueberry Gall Midge, Dasineura oxycoccana (Diptera: Cecidomyiidae), on Blueberry and Cranberry and Testing Invasion Scenarios
Source: Insects. 2022 Sep 28;13(10):880. doi: 10.3390/insects13100880 (PMC9604482; doi:10.3390/insects13100880)
Supplement: Supplementary file 1 [file insects-13-00880-s001.zip › Supplementary material 2 Tables S1-S4.pdf]

Population Genetics of the Blueberry Gall Midge, *Dasineura oxycoccana* (Diptera: Cecidomyiidae), on Blueberry and Cranberry and Testing Invasion Scenarios

Hyojoong Kim [1,*], Cesar Rodriguez-Saona [2,*] and Heung-Sik Lee [3]

[1] Animal Systematics Laboratory, Department of Biological Science, Kunsan National University, Gunsan, Jeonbuk 54150, Korea
[2] Department of Entomology, P.E. Marucci Center, Rutgers University, Chatsworth, NJ 08019, USA
[3] Animal & Plant Quarantine Agency, Gimcheon, Gyeongbuk 39660, Korea
* Correspondence: hkim@kunsan.ac.kr (H.K.); crodriguez@aesop.rutgers.edu (C.R.-S.)

Supplementary material 2: Tables S1-S4

Table S1. Collection data for 31 different geographical and/or host-associated populations of *Dasineura oxycoccana* collected from blueberry and cranberry in Korea and US analyzed in this study.

Collection No.	Population ID	Country	Host plant	No. of individuals	Collection site	GPS-N	GPS-E	Date (YYYY-MM-DD)
1	KR-B-UW	KOREA	blueberry	20	Uiwang	37°19'59.8"N	126°58'52.0"E	2013-05-15
2	KR-B-GJ	KOREA	blueberry	17	Gwangju	37°28'03.4"N	127°20'14.3"E	2012-09-27
3	KR-B-HS1	KOREA	blueberry	23	Hwaseong	37°14'13.8"N	126°42'46.7"E	2012-06-26
4	KR-B-HS2	KOREA	blueberry	12	Hwaseong	37°09'59.0"N	126°49'37.5"E	2012-06-13
5	KR-B-KY	KOREA	blueberry	20	Koyang	37°41'34.7"N	126°42'23.4"E	2012-07-11
6	KR-B-PT	KOREA	blueberry	2	Pyeongtataek	36°59'54.6"N	127°04'00.4"E	2012-10-18
7	KR-B-HE	KOREA	blueberry	18	Hweongseong	37°27'02.4"N	128°03'00.7"E	2012-09-27
8	KR-B-CW	KOREA	blueberry	20	Cheongwon	36°44'38.2"N	127°33'23.3"E	2012-06-28
9	KR-B-YD	KOREA	blueberry	39	Yeongdong	36°05'22.6"N	127°40'21.7"E	2012-05-24
10	KR-B-DA	KOREA	blueberry	15	Dangjin	36°51'11.4"N	126°33'09.4"E	2012-03-23
11	KR-B-CA	KOREA	blueberry	20	Cheonan	36°57'18.3"N	127°11'12.9"E	2012-06-28
12	KR-B-DJ	KOREA	blueberry	9	Daejeon	36°16'05.3"N	127°19'03.0"E	2012-09-28
13	KR-B-YS	KOREA	blueberry	8	Yesan	36°35'11.7"N	126°51'09.1"E	2012-05-29
14	KR-B-IS	KOREA	blueberry	20	Imsil	35°35'44.4"N	127°16'00.2"E	2013-06-05
15	KR-B-SC	KOREA	blueberry	20	Sunchang	35°30'19.1"N	127°00'09.1"E	2012-07-03
16	KR-B-HW	KOREA	blueberry	20	Hwasun	34°52'24.3"N	127°02'27.0"E	2012-07-04
17	KR-B-BH1	KOREA	blueberry	13	Bonghwa1	36°50'24.4"N	128°46'04.2"E	2013-05-19
18	KR-B-BH2	KOREA	blueberry	7	Bonghwa2	36°50'24.4"N	128°46'04.2"E	2013-05-19
19	KR-B-SJ	KOREA	blueberry	31	Sangju	36°32'22.2"N	128°03'28.6"E	2011-08-10
20	KR-B-NH	KOREA	blueberry	20	Namhae	34°49'00.4"N	127°55'36.0"E	2012-07-04
21	KR-B-JJ1	KOREA	blueberry	11	Jeju	33°21'60.0"N	126°21'20.7"E	2013-07-31
22	KR-B-JJ2	KOREA	blueberry	19	Jeju	33°27'55.4"N	126°24'29.2"E	2013-07-10
23	US-B-GA1	USA	blueberry	8	Georgia	31°02'22.6"N	82°55'55.8"W	2012-07-06
24	US-B-GA2	USA	blueberry	9	Georgia	31°12'17.5"N	82°28'28.2"W	2012-07-07
25	US-B-NJ1	USA	blueberry	4	New Jersey	39°54'59.7"N	74°35'48.7"W	2012-07-03
26	US-B-NJ2	USA	blueberry	32	New Jersey	39°35'24.3"N	74°46'00.4"W	2012-07-04
27	US-B-NJ3	USA	blueberry	35	New Jersey	39°42'56.6"N	74°30'59.1"W	2012-07-04
28	US-B-MG	USA	blueberry	40	Michigan	42°50'23.2"N	86°09'46.9"W	2013-06-20
29	US-C-NJ4	USA	cranberry	40	New Jersey	39°42'56.6"N	74°30'59.1"W	2013-05-30

30	US-C-MA	USA	cranberry	40	Massachusetts	41°52'42.2"N	70°43'47.1"W	2013-05-23
31	US-C-WC	USA	cranberry	40	Wisconsin	44°22'59.9"N	89°52'17.9"W	2013-07-23

Table S2. Pairwise F_{ST} divergence between 31 different geographical and/or host-associated populations of *Dasineura oxycoccana* estimated by ARLEQUIN. Values are significantly different at significant level, $P = 0.001$ unless ($P < 0.001$) indicated as bold and underlined.

	KR-B-UW	KR-B-GJ	KR-B-HS1	KR-B-HS2	KR-B-KY	KR-B-PT	KR-B-HE	KR-B-CW	KR-B-YD	KR-B-DA	KR-B-CA	KR-B-DJ	KR-B-YS	KR-B-IS	KR-B-SC	KR-B-HW	KR-B-BH1	KR-B-BH2	KR-B-SJ	KR-B-NH	KR-B-JJ1	KR-B-JJ2	US-B-GA1	US-B-GA2	US-B-NJ1	US-B-NJ2	US-B-NJ3	US-B-MG	US-C-NJ4	US-C-MA
KR-B-GJ	0.092																													
KR-B-HS1	0.059	0.075																												
KR-B-HS2	0.097	0.006	0.057																											
KR-B-KY	0.019	0.085	0.064	0.107																										
KR-B-PT	0.128	**-0.024**	0.113	0.054	0.117																									
KR-B-HE	0.202	0.121	0.223	0.172	0.213	0.161																								
KR-B-CW	0.013	0.051	0.034	0.072	0.009	0.102	0.202																							
KR-B-YD	0.024	0.093	0.052	0.104	0.031	0.113	0.217	0.036																						
KR-B-DA	0.058	0.020	0.028	0.012	0.044	0.078	0.192	0.030	0.064																					
KR-B-CA	0.168	0.094	0.193	0.140	0.190	0.147	0.033	0.175	0.189	0.162																				
KR-B-DJ	0.027	0.148	0.107	0.134	0.047	0.175	0.244	0.049	0.072	0.077	0.228																			
KR-B-YS	0.064	**-0.018**	0.071	0.042	0.054	0.034	0.165	0.021	0.079	0.013	0.138	0.096																		
KR-B-IS	**0.006**	0.118	0.061	0.125	**0.008**	0.161	0.231	0.015	0.032	0.067	0.206	0.041	0.078																	
KR-B-SC	0.017	0.043	0.043	0.054	0.020	0.108	0.173	**0.001**	0.031	0.020	0.142	0.067	0.025	0.029																
KR-B-HW	0.013	0.086	0.040	0.078	0.031	0.120	0.222	0.014	0.033	0.034	0.193	0.027	0.040	0.020	0.020															
KR-B-BH1	0.115	0.072	0.152	0.117	0.136	0.123	0.177	0.111	0.114	0.118	0.144	0.176	0.082	0.145	0.101	0.108														
KR-B-BH2	0.157	0.084	0.173	0.135	0.172	0.104	0.094	0.154	0.164	0.143	0.036	0.216	0.116	0.191	0.140	0.176	0.141													
KR-B-SJ	0.102	**-0.033**	0.084	0.022	0.082	**0.002**	0.150	0.073	0.096	0.033	0.131	0.131	0.006	0.109	0.061	0.091	0.078	0.118												
KR-B-NH	0.052	0.095	0.062	0.112	0.031	0.103	0.236	0.033	0.053	0.059	0.213	0.077	0.065	0.053	0.062	0.049	0.153	0.190	0.101											
KR-B-JJ1	0.056	0.096	0.089	0.121	0.058	**0.080**	0.178	0.050	0.042	0.087	0.163	0.091	0.079	0.061	0.057	0.061	0.079	0.134	0.095	0.064										
KR-B-JJ2	0.241	0.155	0.253	0.228	0.261	0.193	0.227	0.239	0.242	0.238	0.209	0.298	0.210	0.266	0.239	0.269	0.191	0.201	0.198	0.275	0.246									
US-B-GA1	0.200	0.158	0.236	0.212	0.225	0.181	0.214	0.212	0.211	0.219	0.196	0.261	0.196	0.232	0.204	0.238	0.204	0.194	0.183	0.250	0.222	0.115								
US-B-GA2	0.195	0.103	0.203	0.154	0.227	0.141	0.203	0.196	0.210	0.178	0.165	0.269	0.165	0.241	0.181	0.222	0.196	0.184	0.142	0.235	0.230	0.142	0.134							
US-B-NJ1	0.078	0.087	0.132	0.124	0.116	0.121	0.120	0.091	0.108	0.115	0.107	0.120	0.087	0.102	0.082	0.106	0.082	0.089	0.104	0.123	0.085	0.170	0.142	0.166						
US-B-NJ2	0.091	0.052	0.117	0.096	0.122	0.077	0.128	0.099	0.107	0.096	0.111	0.127	0.081	0.114	0.088	0.109	0.087	0.095	0.087	0.129	0.090	0.178	0.150	0.161	0.046					
US-B-NJ3	0.098	0.060	0.102	0.094	0.118	0.090	0.137	0.098	0.105	0.085	0.125	0.128	0.078	0.115	0.081	0.111	0.104	0.112	0.087	0.141	0.098	0.186	0.166	0.165	0.077	0.021				
US-B-MG	0.167	0.104	0.196	0.158	0.187	0.148	0.052	0.174	0.185	0.166	0.036	0.222	0.141	0.195	0.151	0.192	0.123	0.060	0.136	0.213	0.156	0.185	0.171	0.169	0.095	0.099	0.115			
US-C-NJ4	0.257	0.183	0.272	0.247	0.266	0.223	0.243	0.259	0.255	0.255	0.221	0.307	0.235	0.281	0.258	0.284	0.214	0.209	0.217	0.288	0.257	0.169	0.222	0.265	0.222	0.195	0.196	0.209		
US-C-MA	0.279	0.213	0.306	0.285	0.300	0.273	0.268	0.291	0.283	0.289	0.242	0.339	0.249	0.300	0.272	0.308	0.245	0.265	0.238	0.336	0.303	0.229	0.267	0.301	0.245	0.220	0.213	0.235	0.121	
US-C-WC	0.329	0.256	0.351	0.333	0.355	0.339	0.308	0.340	0.328	0.340	0.278	0.398	0.305	0.352	0.325	0.357	0.279	0.309	0.281	0.379	0.356	0.238	0.310	0.336	0.290	0.257	0.252	0.272	0.121	0.076

Table S3. Results of the bottleneck test based on the two mutation models, SMM and TPM, using a nonparametric Wilcoxon signed-rank test. Values in bold indicate detection of the genetic bottleneck ($P < 0.001$, one tail for heterozygote excess).

Pop. ID	No.	Wilcoxon signed-rank tests		Mode shift
		TPM	SMM	
KR-B-UW	20	0.42505	0.95386	normal
KR-B-GJ	17	0.99768	1.00000	normal
**KR-B-HS1**	**23**	**0.01709**	**0.04614**	**normal**
KR-B-HS2	12	0.68896	0.94507	normal
KR-B-KY	20	0.15063	0.78809	normal
KR-B-PT	2	n/a	n/a	n/a
KR-B-HE	18	0.15063	0.98291	normal
KR-B-CW	20	0.25928	0.96143	normal
KR-B-YD	39	0.95386	0.99988	normal
KR-B-DA	15	0.21191	0.66138	normal
KR-B-CA	20	0.05493	0.80981	normal
KR-B-DJ	9	0.04614	0.42505	shifted
KR-B-YS	8	n/a	n/a	n/a
KR-B-IS	20	0.42505	0.97876	normal
KR-B-SC	20	0.63330	0.97388	normal
**KR-B-HW**	**20**	**0.00232**	**0.04614**	**shifted**
KR-B-BH1	13	0.91187	0.99829	normal
KR-B-BH2	7	0.02612	0.23486	normal
KR-B-SJ	31	0.36670	0.99915	normal
KR-B-NH	20	0.13306	0.57495	normal
KR-B-JJ1	11	0.48486	0.89819	normal
KR-B-JJ2	19	0.39551	0.95386	normal
US-B-GA1	8	0.03857	0.16968	shifted
US-B-GA2	9	0.45483	0.78809	normal
US-B-NJ1	4	0.23486	0.51514	shifted
US-B-NJ2	32	0.95386	0.99988	normal
US-B-NJ3	35	0.74072	0.98291	normal
US-B-MG	40	0.83032	0.99939	normal
US-C-NJ4	40	0.97388	0.99939	normal

US-C-MA	40	0.76758	0.99390	normal
US-C-WC	40	0.96802	0.99695	normal

Table S4. Mean assignment rate of 632 *Dasineura oxycoccana* individuals of 31 different geographical and/or host-associated populations into (rows) and from (columns) each population using GENECLASS2. Values in bold and underline indicate the proportions of individuals assigned to the source population (i.e. self-assignment). Values in bold and red indicate the proportions of individuals assigned to the most likely source except for the self-assignment (i.e. the most likely origin). Zero values were excluded from the table.

	KR-B-UW	KR-B-GJ	KR-B-HS1	KR-B-HS2	KR-B-KY	KR-B-PT	KR-B-HE	KR-B-CW	KR-B-YD	KR-B-DA	KR-B-CA	KR-B-DJ	KR-B-YS	KR-B-IS	KR-B-SC	KR-B-HW
**KOREA**																
KR-B-UW	**0.493**	0.010			0.126			0.128	0.227	0.024		0.031	0.033	**0.318**	0.175	
KR-B-GJ	0.008	**0.572**	0.017	0.098	0.011	0.302		0.038	0.021	0.095			0.153		0.071	
KR-B-HS1	0.345	0.122	**0.499**	0.391	0.126	0.015		0.441	0.287	**0.445**			0.233	0.292	0.400	0.108
KR-B-HS2	0.023	**0.350**	0.012	**0.319**	0.006	0.027		0.048	0.017	0.114			0.221		0.158	
KR-B-KY	0.540	0.093	0.054	0.078	**0.481**			**0.561**	0.468	0.153		0.071	0.225	0.488	0.542	0.094
KR-B-PT		**0.769**		0.063		**0.000**							0.346		0.037	
KR-B-HE							**0.470**				0.183		0.000			
KR-B-CW	0.275	0.065	0.023	0.070	0.200			**0.425**	0.260	0.132		0.020	0.296	0.237	**0.432**	0.031
KR-B-YD	**0.477**	0.029	0.059	0.033	0.210			0.279	**0.568**	0.080		0.040	0.080	0.357	0.377	0.109
KR-B-DA	0.176	0.216	0.138	0.302	0.111	0.011		0.205	0.148	**0.396**		0.017	0.252	0.146	0.269	0.113
KR-B-CA							0.058				**0.388**					
KR-B-DJ	**0.708**	0.058	0.016	0.079	0.241			0.445	0.373	0.082		**0.469**	0.146	0.505	0.380	0.023
KR-B-YS	0.044	**0.408**	0.016	0.171	0.070	0.116		0.189	0.055	0.120			**0.316**	0.026	0.266	
KR-B-IS	**0.573**	0.013	0.038	0.015	0.212			0.226	0.323	0.051		0.033	0.053	**0.503**	0.230	0.054
KR-B-SC	**0.272**	0.088		0.052	0.129	0.021		0.287	0.256	0.092		0.008	0.146	0.168	**0.451**	0.012
KR-B-HW	**0.671**	0.131	0.176	0.176	0.423	0.015		0.590	0.572	0.301		0.064	0.348	0.571	0.584	**0.473**
KR-B-BH1		0.067		0.015		0.083			0.011				0.062			
KR-B-BH2											0.075					
KR-B-SJ		**0.411**		0.130	0.006	0.084		0.013	0.019	0.046			0.179	0.004	0.064	
KR-B-NH	0.452	0.122	0.075	0.118	0.297			0.445	0.446	0.233		0.042	0.224	0.358	**0.455**	0.123
KR-B-JJ1	0.104	0.021		0.010	0.026			0.044	**0.174**			0.005	0.023	0.075	0.108	
KR-B-JJ2																
**USA**																
US-B-GA1																
US-B-GA2																
US-B-NJ1																
US-B-NJ2																
US-B-NJ3																
US-B-MG																
US-C-NJ4																
US-C-MA																
US-C-WC																

	KR-B-BH1	KR-B-BH2	KR-B-SJ	KR-B-NH	KR-B-JJ1	KR-B-JJ2	US-B-GA1	US-B-GA2	US-B-NJ1	US-B-NJ2	US-B-NJ3	US-B-MG	US-C-NJ4	US-C-MA	US-C-WC
**KOREA**															
KR-B-UW			0.019	0.011	0.143				0.068	0.025					
KR-B-GJ	0.027		**0.349**					0.027	0.097	0.041	0.034				
KR-B-HS1			0.274	0.081	0.089				0.067	0.114	0.050				
KR-B-HS2	0.014		0.404						0.058	0.077	0.041				
KR-B-KY	0.025		0.223	0.142	0.255				0.090	0.160	0.031				
KR-B-PT	0.027		0.293						0.065	0.044					
KR-B-HE		0.054							0.038			**0.474**			
KR-B-CW			0.115	0.039	0.139				0.100	0.183	0.057				
KR-B-YD	0.003		0.041	0.091	0.307				0.066	0.091	0.038				
KR-B-DA	0.023		**0.287**	0.122	0.071				0.060	0.184	0.051				
KR-B-CA		0.165							0.029			**0.509**			
KR-B-DJ			0.181	0.011	0.194				0.132	0.255	0.081				
KR-B-YS	0.048	0.012	0.359	0.010	0.012				0.143	0.168	0.038				
KR-B-IS			0.025	0.067	0.241				0.078	0.060					
KR-B-SC	0.011		0.101	0.032	0.120				0.088	0.140	0.082				
KR-B-HW	0.014		0.226	0.164	0.241				0.167	0.260	0.052				
KR-B-BH1	**0.394**		0.023						**0.085**	0.014					
KR-B-BH2		**0.241**							0.019			**0.214**			
KR-B-SJ	0.026		**0.496**						0.054	0.064	0.030				
KR-B-NH			0.176	**0.499**	0.235				0.117	0.177	0.020				
KR-B-JJ1	0.025		0.016		**0.299**				0.092	0.058	0.011				
KR-B-JJ2						**0.383**			0.029						
**USA**															
US-B-GA1							**0.288**								
US-B-GA2								**0.348**	0.010						
US-B-NJ1									**0.080**	0.103					
US-B-NJ2									0.049	**0.344**	**0.150**				
US-B-NJ3									0.020	**0.230**	**0.419**				
US-B-MG		**0.043**							0.043			**0.489**			
US-C-NJ4									0.019				**0.492**	0.017	**0.022**
US-C-MA									0.027				**0.168**	**0.506**	0.041
US-C-WC									0.038				0.262	**0.268**	**0.580**
